# Fluorescence‐based discrimination of breast cancer cells by direct exposure to 5‐aminolevulinic acid

**DOI:** 10.1002/cam4.2466

**Published:** 2019-08-05

**Authors:** Midori Morita, Hideo Tanaka, Yasuaki Kumamoto, Akihiro Nakamura, Yoshinori Harada, Takehiro Ogata, Koichi Sakaguchi, Tetsuya Taguchi, Tetsuro Takamatsu

**Affiliations:** ^1^ Department of Pathology and Cell Regulation, Graduate School of Medical Science Kyoto Prefectural University of Medicine Kyoto Japan; ^2^ Department of Endocrine and Breast Surgery, Graduate School of Medical Science Kyoto Prefectural University of Medicine Kyoto Japan; ^3^ Department of Medical Photonics Kyoto Prefectural University of Medicine Kyoto Japan

**Keywords:** 5‐aminolevulinic acid, ATP‐binding cassette transporter G2, breast cancer, fine needle aspiration cytology, photodynamic diagnosis

## Abstract

Protoporphyrin IX‐fluorescence measurement is a powerful in situ approach for cancer detection after oral/topical administration of 5‐aminolevulinic acid. However, this approach has not been clinically established for breast cancer, probably due to insufficient delivery of 5‐aminolevulinic acid to the mammary glands. In the present study, we directly exposed breast cancer cells to 5‐aminolevulinic acid to assess their discrimination via protoporphyrin IX‐fluorescence. Fluorescence intensity (FI) was measured in the human breast cancer cell lines MCF7 and MDA‐MB‐231 and breast epithelial cell line MCF10A by confocal microscopy and flow cytometry. After 5‐aminolevulinic acid exposure for 2 hours, protoporphyrin IX‐FI in MCF7 and MDA‐MB‐231 cells significantly increased with marked cell‐to‐cell variability, whereas that in MCF10A cells increased moderately. Combined exposure of the cancer cells to 5‐aminolevulinic acid and Ko143, a specific inhibitor of ATP‐binding cassette transporter G2, further increased protoporphyrin IX‐FI and alleviated the cell‐to‐cell variability in MCF7 and MDA‐MB‐231 cells, indicating improvement in the reproducibility and accuracy for fluorescence‐based cancer detection. The increased FI by combined administration of these two drugs was also demonstrated in cells obtained via fine needle aspiration from mouse xenograft models inoculated with MDA‐MB‐231 cells. Furthermore, a cutoff value for increased protoporphyrin IX‐FI ratio, before and after exposure to these drugs, clearly discriminated between cancer and noncancer cells. Taken together, direct exposure to 5‐aminolevulinic acid and Ko143 may be a promising strategy for efficient fluorescence‐based detection of breast cancer cells ex vivo using fine needle aspiration.

## 1 INTRODUCTION

Breast cancer is the most frequently occurring malignant neoplasm in women worldwide, and more than 2 million women are newly diagnosed with breast cancer each year.^1^ It is one of the leading causes of cancer‐related deaths in women.^1^, ^2^ Early detection and accurate diagnosis are essential to improve outcomes and survival of patients with breast cancer.

Regarding the pathological diagnosis of breast cancer, fine needle aspiration (FNA) cytology (FNAC) has been widely used for primary screening^3^; however, despite its well accepted and classical means, its diagnostic validity has been limited due to the absence of objective or definitive evidence for cells being cancer or noncancer. The interpretation of FNAC samples depends entirely upon the cytopathologists’ subjective judgments based on certain empirical diagnostic criteria,^4^ where sensitivity and specificity of breast cancer diagnosis are variables ranging from 35% to 95% and from 48% to 100%, respectively.^5^ In this respect, innovative strategies are warranted for cancer screening in FNAC, that is, more definitive and reliable discrimination methods for cancer cells, vs conventional cytology or histopathology of needle aspiration/biopsy samples.

For more than two decades, fluorescence imaging has emerged as a powerful tool for efficient detection of malignant neoplasms in situ.^6^, ^7^, ^8^, ^9^ Among various chemical compounds for the fluorescence approach, 5‐aminolevulinic acid (5‐ALA) has been extensively tested in experimental and clinical studies.^9^, ^10^, ^11^, ^12^, ^13^ Orally or topically administered 5‐ALA accumulates the fluorescence metabolite protoporphyrin IX (PpIX) within cancer cells due to their relatively higher activity of porphobilinogen deaminase^14^ and/or lower activity of ferrochelatase^15^, ^16^, ^17^, ^18^ compared to noncancer cells. Clinically, the PpIX‐fluorescence–based detection of cancer has been established mostly in the intraoperative settings of brain and urinary bladder tumors.^12^, ^19^, ^20^ However, the efficacy of 5‐ALA for detecting PpIX in breast cancer is currently undetermined. Previous studies reported large variations in PpIX‐fluorescence intensity (PpIX‐FI) among patients after oral intake of 5‐ALA,^13^, ^21^ presumably because of the complex pharmacokinetics and metabolism of 5‐ALA in situ^22^, ^23^, ^24^ and the influence of autofluorescence within human tissues (eg, flavin adenine dinucleotide).^25^ To exclude these ambiguous factors for the breast cancer in situ, direct exposure of cell specimens ex vivo to 5‐ALA may help to detect PpIX‐fluorescence.

Our hypothesis is that administration of 5‐ALA directly to cells may provide accurate discrimination between cancer and noncancer cells through PpIX‐fluorescence detection. The present study assessed the effect of direct exposure to 5‐ALA on PpIX accumulation in human breast cancer cell lines in vitro. We also evaluated this approach's applicability to ex vivo specimens obtained through FNA from mouse xenografts of a breast cancer cell line. Furthermore, we investigated the role of ATP‐binding cassette transporter G2 (ABCG2) on PpIX‐fluorescence, which may influence the intracellular transport of PpIX efflux.

## 2 MATERIALS AND METHODS

### 2.1 Cell culture

We used two representative types of human breast cancer cell lines, that is, MCF7 (Public Health England) and MDA‐MB‐231 (American Type Culture Collection; ATCC), which are, respectively, responsive and nonresponsive to estrogen, and the human breast epithelial cell line MCF10A (ATCC). All cell lines were cultured in DMEM supplemented with 10% fetal bovine serum and 1% antibiotics and antimycotics.

### 2.2 Chemicals

We used 5‐ALA hydrochloride (COSMO BIO), which was dissolved into DMEM at various concentrations. In some experiments, we used the ABCG2 transporter inhibitor Ko143 (Sigma‐Aldrich), which were stored at −20°C after dissolution in DMSO. Polyclonal antibodies against ABCG2 (Cell Signaling Technology) and peptide transporter 1 (PEPT1) were purchased from Bioss Inc. The bicinchoninic acid protein assay kit was obtained from Wako Pure Chemicals.

### 2.3 Establishment of stable transfectants

A total of 4 μg of pEGFP/C1 (Clontech Laboratories) was transfected to MDA‐MB‐231 cells with lipofectin (Invitrogen) according to the manufacturer's instructions. The cells that expressed green fluorescence protein (GFP) stably were selected in the presence of 1 mg/mL of G‐418 (Nacalai tesque). Successful expression of the GFP gene was confirmed by fluorescence microscopy.

### 2.4 Confocal fluorescence microscopic imaging and analysis

For confocal fluorescence microscopic imaging, we seeded 2 × 10^5^ cells on plastic dishes (35‐mm diameter) and cultured for 2 days. The cells were subsequently incubated for 2 hours in DMEM containing 5‐ALA (5 mmol/L). After incubation with 5‐ALA, the cells were immersed in fresh culture medium. The cells were excited at the wavelength of 440 nm (*λ*_ex_) for PpIX‐fluorescence imaging at the wavelength of 615‐645 nm (*λ*_em_) by a confocal laser scanning system (FV1000; Olympus) equipped with an inverted microscope (IX81, Olympus). Confocal fluorescence imaging was conducted via 20X objective lens (NA = 0.75) under a confocal aperture of 160 μm with the imaging field of view was 636 × 636 μm (1024 × 1024 pixels). Through the experiment, we kept all the measurement conditions constant, that is, photomultiplier voltage, acquisition time, and excitation light intensity.

Fluorescence intensity was analyzed using the NIH ImageJ software (Ver. 1.51, 16 bits) as described by Millon et al^25^ The mean FI values of the individual cells (n = 60) were measured from three distinct areas after subtraction of the background FI of cell‐free area.

### 2.5 Optimal dose and duration for cancer discrimination

To distinguish between cancer and noncancer cells, we determined the optimized dose and duration of exposure for 5‐ALA (data not shown). Based on preliminary experiments, we set the dose and exposure durations of 5‐ALA to 5 mmol/L and 2 hours, respectively.

### 2.6 Flow cytometry analysis

Cells were seeded at a density of 2 × 10^5^ cells in 35‐mm dishes. After incubation for 2 days, cells were incubated with or without 5‐ALA (5 mmol/L) for 2 hours. The cells were subsequently rinsed with PBS, trypsinized, and suspended in fresh DMEM. PpIX‐fluorescence was measured using the cell sorter SH800S (SONY) in the FL4 channel (*λ*_ex_, 405 nm; *λ*_em_, 600‐685 nm). Of a total of 1 × 10^5^ cells served for the cytometry, 3‐5 × 10^4^ cells were identified by first gating on live cells (FSC‐A vs BSC‐A) and on singlets (FSC‐H vs FSC‐W) for each experiment. In some experiments, the mean FI values of 5‐ALA–induced PpIX‐FI were obtained by subtracting the FI of cells without 5‐ALA from the FI of cells with 5‐ALA (Figure 5B and C).

### 2.7 Biochemical study

Cells washed with PBS twice were lysed using Leammli's buffer (25 mmol/L Tris‐HCl, 2% SDS, 10% glycerol, pH = 6.8) lacking the tracking dye and reducing reagent. After DNA shearing, extracted cellular proteins were reduced and processed for immunoblotting. In brief, the blots were blocked using 3% BSA‐TBS‐T (0.15 mol/L NaCl, 0.05% Tween 20, 10 mmol/L Tris‐HCl), termed “blocking buffer,” at room temperature. The treated blots were incubated with blocking buffer containing anti‐PEPT‐1 antibody (1:2000) (Bioss Inc) or anti‐ABCG2 antibody (1:1000) (CST) at 4°C overnight. Subsequently, the blots were incubated with blocking buffer containing HRP‐labeled anti‐rabbit IgG antibody (1:4000) (Cayman Chemical) at room temperature for 1 hour. The treated blots were developed using the Immunostar Zeta (WAKO). After immunodetection, the blots were stained using protein staining solution (0.1% Coomassie brilliant Blue R‐250, 30% methanol, 10% acetic acid), followed by destaining with 30% methanol and 10% acetic acid. Chemiluminescence signals were calibrated to the total protein staining.^26^, ^27^ Statistical significance was evaluated using Bonferroni's multiple comparison method.

### 2.8 Tumor‐bearing mouse model

Female BALB/c nude mice (5‐8 weeks old) were inoculated subcutaneously with MDA‐MB‐231 (1 × 10^7^ cells) stably expressing EGFP. After inoculation, the mice were regularly fed. All animal experiments were approved by the institutional guidelines of Kyoto Prefectural University of Medicine.

### 2.9 Fluorescence detection protocol for fine needle aspiration specimens obtained from a breast cancer xenograft

Approximately 2 months after inoculation with MDA‐MB‐231 cells, specimens from subcutaneous nodules were extracted through FNA using 20‐mm syringes (21 gage syringe needle) and maintained in culture medium without 5‐ALA and Ko143. Harvested cells were divided into two tubes (ie, with and without combined 5‐ALA and Ko143 exposure) and subsequently incubated for 2 hours at 37°C to allow the production and accumulation of intracellular PpIX. After the co‐administration of 5‐ALA (5 mmol/L) and Ko143 (1 μmol/L), subsequent procedures were performed under light‐shielded conditions. The cell suspension was centrifuged for 5 minutes at 400 *g* and the cell pellet was washed using PBS. After centrifugation, cells were trypsinized and suspended in fresh DMEM. The PpIX‐FI was measured using SH800S cell sorter or FV1000 confocal microscopy.

### 2.10 Statistical analysis

All experiments were performed in triplicate and quantitative data were expressed as mean ± SD. Unless specified otherwise, statistical analyses were performed by the unpaired Student's *t* test (*P* < .05). In some experiments, statistical analyses were performed using the unpaired Student's *t* test, followed by Bonferroni's correction.

## 3 RESULTS

### 3.1 Detection of PpIX‐fluorescence in 5‐ALA–treated cells

**Figure 1:**
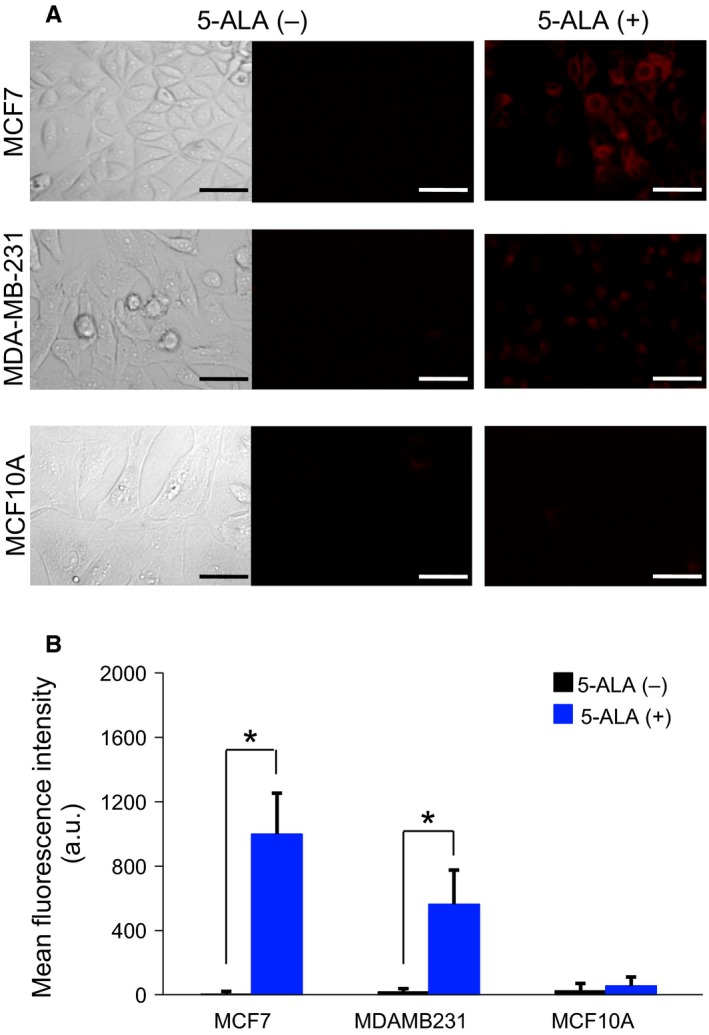
Confocal fluorescence microscopic analyses of MCF7, MDA‐MB‐231, and MCF10A cells before and after treatment with 5‐ALA. (A), Representative confocal images of PpIX‐fluorescence (*λ*_ex_: 440 nm; *λ*_em_: 615‐645 nm) before and 2 hours after 5 mmol/L 5‐ALA exposure in association with the differential interference contrast images (left panels). Bars = 50 μm. (B), Mean fluorescence intensity (FI) of 5‐ALA–induced PpIX (mean ± SD) after a 2‐h incubation (n = 3). *P < .05

Direct exposure to 5‐ALA resulted in marked enhancement of red PpIX‐fluorescence in breast cancer cell lines MCF7 and MDA‐MB‐231. When compared with the red fluorescence before and after 5‐ALA exposure, the red PpIX‐FI measured by confocal microscopy was relatively high in comparison to the indiscernible or low‐grade red autofluorescence after exposure (Figure 1A). Quantitatively, the mean PpIX‐FI after 5‐ALA exposure was significantly higher in MCF7 and MDA‐MB‐231 cells than the mean PpIX‐FI before treatment (*P* < .05). However, the mean PpIX‐FI in MCF10A showed no statistically significant increments by 5‐ALA (Figure 1B).

**Figure 2:**
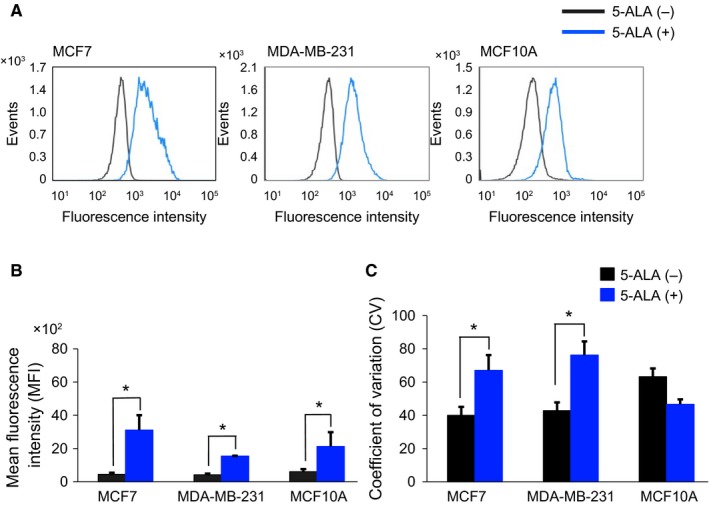
Flow cytometric analyses of 5‐ALA–induced PpIX‐FI for MCF7, MDA‐MB‐231, and MCF10A cells. (A), Representative histograms of autofluorescence intensity and 5‐ALA–induced PpIX‐FI after 2 h of exposure. (B), Mean FI of 5‐ALA–induced PpIX (mean and SD) after 2 h of incubation. (C), Coefficient of variation (CV) for 5‐ALA–induced PpIX‐FI after 2 h of incubation. All experiments were performed in triplicate. *P < .05 compared with the corresponding autofluorescence

In addition to the direct microscopic visualization of PpIX‐fluorescence of cells, we also performed flow cytometric analysis of the PpIX‐FI in 5‐ALA–treated cells. FI histograms showed significant changes in PpIX‐FI before and after exposure to 5‐ALA for cancer and noncancer cells (Figure 2); the drug markedly shifted the FI histograms rightward along the FI axis (Figure 2A). Quantitatively, the mean FI values obtained through flow cytometry were significantly increased by exposure to 5‐ALA in all three cell lines, that is, in the cancer and noncancer cells (Figure 2B). This result was not completely consistent with the results obtained by confocal microscopy (Figure 1).

Exposure of the two breast cancer cell lines to 5‐ALA increased the mean PpIX‐FI and its coefficient of variation (CV), that is, variability. This effect was not observed in the noncancer cell line MCF10A (Figure 2C). In addition, increments of PpIX‐FI were different between MCF7 and MDA‐MB‐231 (Figures 1B and 2B). Thus, the 5‐ALA–induced intracellular PpIX‐FI varies from cell‐to‐cell and depends on cell lines. The 5‐ALA–induced PpIX‐FI also showed cell‐to‐cell variability for the cancer cell lines by confocal microscopy (Figure 1A).

### 3.2 Expression levels of ABCG2 protein in MCF7, MDA‐MB‐231, and MCF10A

**Figure 3:**
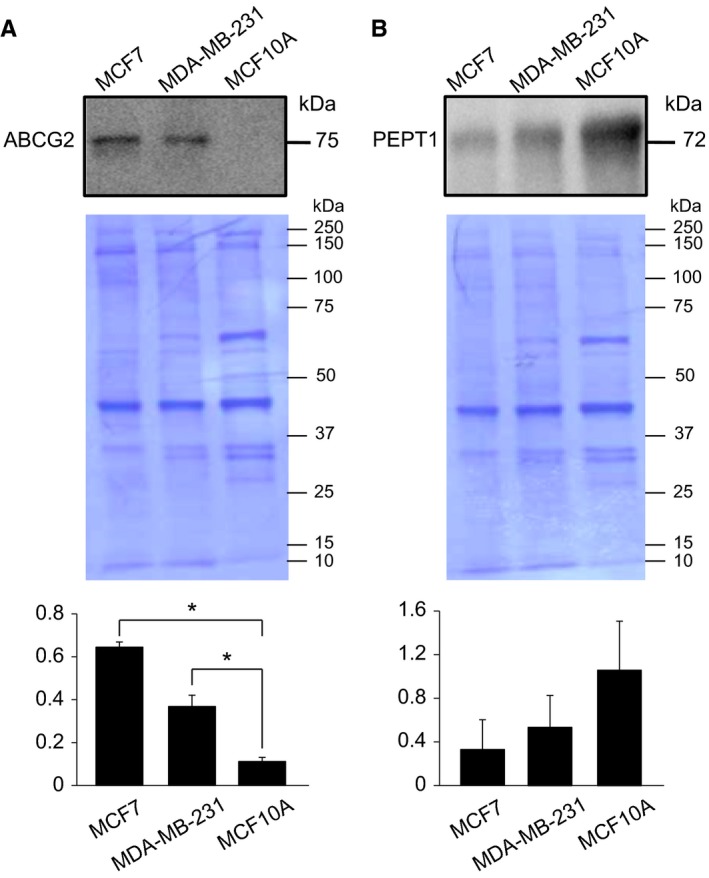
Whole cell lysates were subjected to immunoblotting with anti‐ABCG2 (A, top panel) or PEPT1 antibody (B, top panel), followed by total protein staining (A, B, middle panels). Data are representative of three independent experiments. Graphs present the ratios of signal intensity on the immunoblot to total protein staining (n = 3, mean ± SD). *Statistically significant difference on the basis of Bonferroni's multiple comparison (*P* < .05)

The observed cell‐to‐cell variability of the 5‐ALA–induced PpIX‐FI in cancer cell lines (Figure 1) may be due to differences in intrinsic metabolism of PpIX among cell lines. In addition, the inconsistent results between microscopy and flow cytometry (Figure 1 vs. 2) could be explained by the differences in expressions of membrane transporter proteins. In this respect, there may be differences in the expressions of membrane transporter proteins ABCG2 and PEPT1, which are responsible for the export of PpIX and import of 5‐ALA, respectively.^28^, ^29^, ^30^ In particular, the ABCG2 transporter reportedly plays an important role in the intracellular accumulation of 5‐ALA‐PpIX.^28^ Immunoblots in Figure 3 showed that the levels of ABCG2 protein were significantly higher in MCF7 and MDA‐MB‐231 cells than in MCF10A cells (*P* < .05). In contrast, PEPT1 levels were lower in the two cancer cell lines than those measured in MCF10A cells; however, the difference did not reach statistical significance (*P* = .09 for MCF7; *P* = .18 for MDA‐MB‐231). Based on these observations, the modest increase in PpIX‐FI in MCF10A cells and the cell‐to‐cell variability in MCF7 and MDA‐MB‐231 cells observed by flow cytometry may be, at least in part, attributed to the increased expression of ABCG2 in these cancer cell lines.

### 3.3 Effects of the ABCG2 inhibitor Ko143 on the intensity of 5‐ALA–induced PpIX‐fluorescence

**Figure 4:**
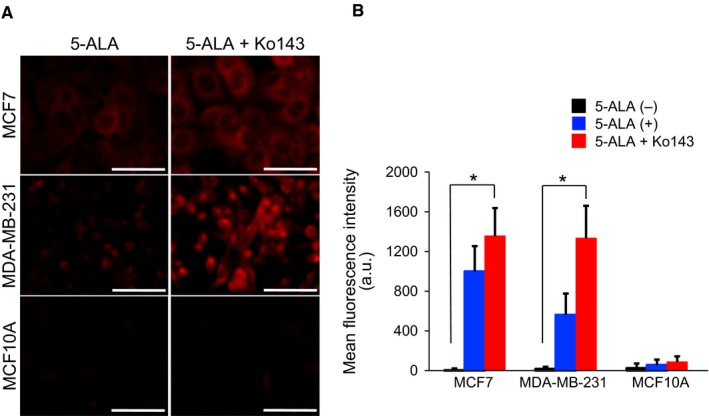
Enhancement of PpIX‐FI with Ko143 in breast cancer cells. (A), Representative confocal fluorescence images of PpIX after incubation with 5‐ALA (5 mmol/L for 2 h) with or without Ko143 (1 μmol/L). Bars = 50 μm. (B), Mean FI of PpIX for 2 h of incubation. All error bars represent SD (n = 3). *P < .05

**Figure 5:**
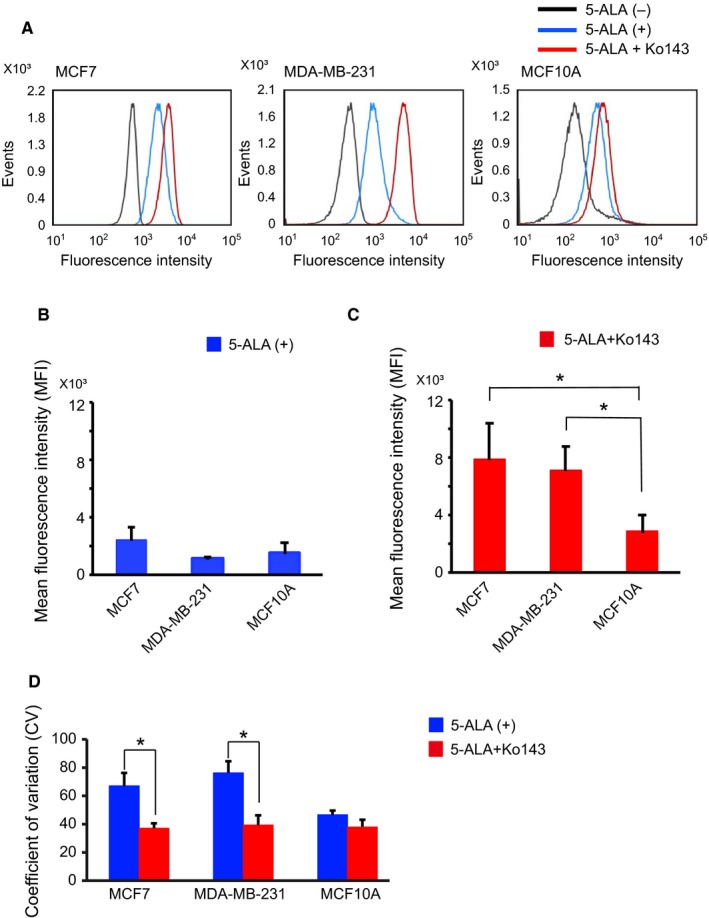
Flow cytometric analysis of Ko143 effect on PpIX‐FI of MCF7, MDA‐MB‐231, and MCF10A cells after 2 h of incubation. (A), Representative histograms of PpIX‐fluorescence with or without exposure to Ko143. (B), Mean FI of 5‐ALA‐PpIX‐FI after subtracting the FI without 5‐ALA. (C), Effects of Ko143 on the mean PpIX‐FI after subtracting the FI without 5‐ALA. *Statistically significant difference on the basis of Bonferroni's multiple comparison (*P* < .05). (D), Effects of Ko143 on the CV of PpIX‐FI. All error bars represent SD (n = 3). *P < .05

An elevated expression level of ABCG2 in the cancer cells can be closely associated with the difficulty in 5‐ALA–based discrimination between cancer (MCF7, MDA‐MB‐231) and noncancer (MCF10A) cell lines in flow cytometry. We therefore tested the effects of Ko143, a synthetic inhibitor for ABCG2, on the 5‐ALA–induced FI values. We used Ko143 at a fixed concentration of 1 μmol/L, based on the preliminary concentration‐dependent responses of Ko143 to 5‐ALA–induced PpIX‐fluorescence (Figure S1). We confirmed that Ko143 per se did not show significant changes in 5‐ALA–induced PpIX‐FI in all three kinds of cell lines (Figure S2). As shown in Figure 4, combined exposure of cells to 5‐ALA and Ko143 significantly increased 5‐ALA‐PpIX‐FI in cancer cell lines under all conditions (*P* < .05). In contrast, the combination treatment induced a modest increase in PpIX‐FI in MCF10A cells (*P* = .056, Figure 4B). Similar results were obtained in the flow cytometric analysis of the combined drug exposure as shown in Figure 5A. This combination significantly increased 5‐ALA‐PpIX‐FI in MCF7 and MDA‐MB‐231 cells compared with MCF10A cells (*P* < .05), whereas the sole 5‐ALA administration without Ko143 showed no difference in 5‐ALA–induced FI between cancer and noncancer cells (Figure 5B and C). Furthermore, the CV of PpIX‐FI, a reflection of cell‐to‐cell variability in PpIX‐FI, was particularly alleviated in the two cancer cell lines (Figure 5C, *P* < .05). Thus, Ko143 enhanced the increase in 5‐ALA‐PpIX‐FI in cancer cell lines and significantly attenuated the cell‐to‐cell variability in response.

### 3.4 Fluorescence analysis on FNA specimens obtained from breast cancer xenografts

**Figure 6:**
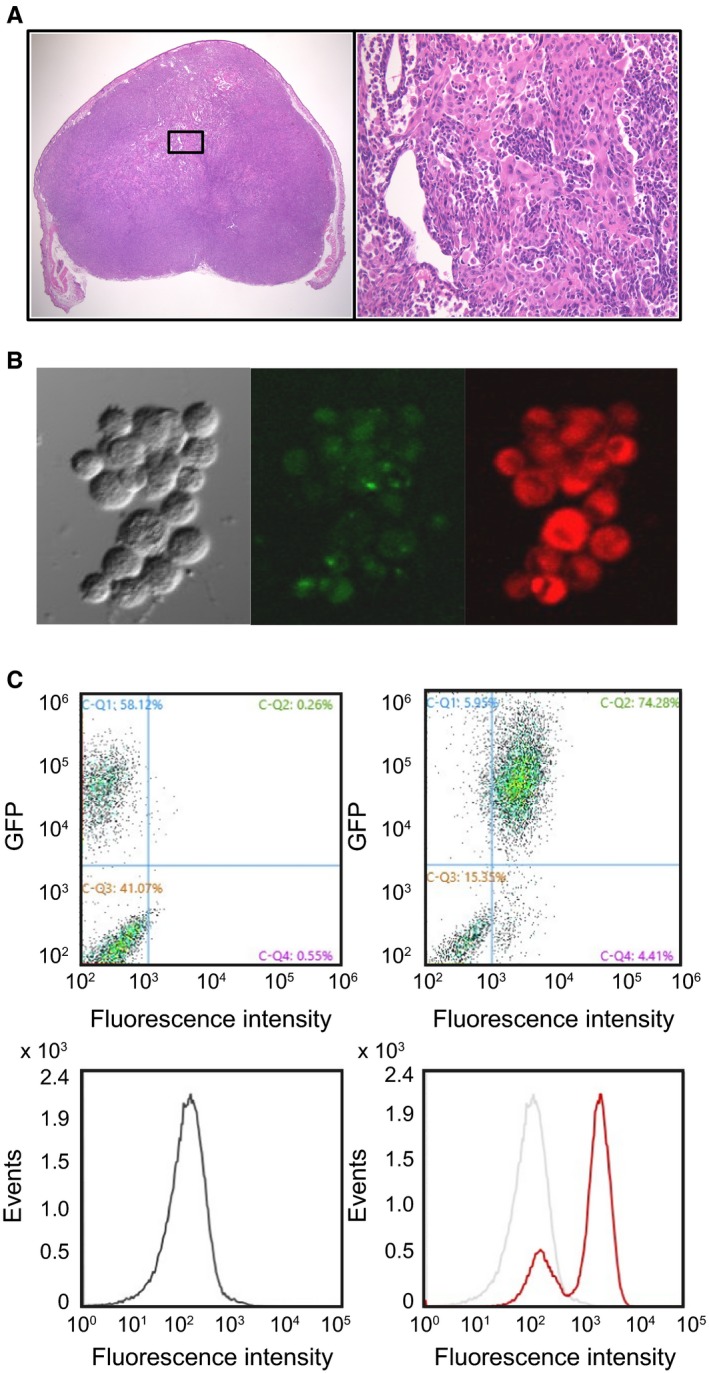
Fluorescent analysis on Fine needle aspiration (FNA) specimens obtained from Enhanced green fluorescent protein (EGFP)‐expressing human breast cancer xenografts. (A), Hematoxylin‐eosin (HE) stained images of a subcutaneous xenograft. (B), Images of EGFP‐expressing MDA‐MB‐231 cells obtained through FNA after exposure to 5‐ALA and Ko143. Differential interference contrast (left), GFP fluorescence (middle) (*λ*_ex_: 488 nm, *λ*_em_: 520‐550 nm), and PpIX‐fluorescence (right) (*λ*_ex_: 440 nm, *λ*_em_: 615‐645 nm) images acquired from the same area. (C), Flow cytometric analyses from the cells with (right) and without (left) exposure to 5‐ALA and Ko143. The histogram indicated by black line denotes the control group (upper left panel). The red line shows the treatment group with 5‐ALA and Ko143 (upper right panel)

We further investigated the effect of exposure to 5‐ALA on increasing PpIX‐fluorescence in cancer cells obtained from 14 subcutaneous xenografts of the breast cancer cell line MDA‐MB‐231 that stably expresses EGFP. As shown in the hematoxylin and eosin (HE)‐stained images, subcutaneous nodules were observed 6‐10 weeks after cell injection (Figure 6A). Confocal fluorescence images revealed that most cells obtained through FNA showed green fluorescence, indicating they were MDA‐MB‐231 cells (Figure 6B). In addition, 5‐ALA–induced PpIX‐FI increased in EGFP‐positive cancer cells. Among the collected populations, flow cytometric analysis also revealed that the EGFP‐positive cell fraction (top left‐hand portion), regarded as a population of cancer cells, shifted to the right after combined exposure to 5‐ALA and Ko143 (Figure 6C). On the other hand, the EGFP‐negative cell fraction (bottom left), considered as a noncancer cell population, barely shifted the fluorescence histogram after exposure to 5‐ALA. Although the EGFP‐labeled cancer cells monotonically enhanced PpIX‐FI, the cytometric FI histogram showed bimodal peaks after combined exposure to 5‐ALA and Ko143, indicating the coexistence of cancer cells and noncancer cells in the xenograft (Figure 6C). Thus, PpIX‐FI was increased in cancer cells obtained from the xenograft through combined exposure to 5‐ALA and Ko143.

### 3.5 Sensitivity of fluorescence cancer detection by FNA

**Figure 7:**
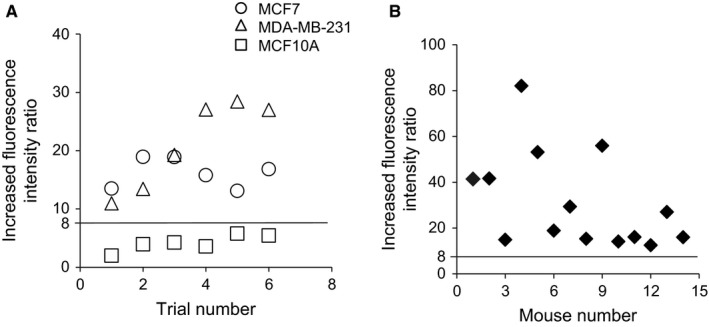
FI ratio between breast cancer cells and noncancer cells after treatment with 5‐ALA and Ko143. (A), The cutoff line at 8, yields 100% sensitivity and 100% specificity for differentiating cancer from noncancer cells. Each value indicates the ratio of the mean FI of cells after the drug exposure over the mean FI without the drug, that is, the autofluorescence intensity. Circles, triangles, and rectangles denote the data of MCF7, MDA‐MB‐231, and MCF10A cells, respectively (n = 6). (B), Plots of the FI ratio of FNAC‐FI from the xenograft (n = 14). All FI ratios of cancer cells were higher than the cutoff value

We evaluated the cancer detectability by FI ratio before and after drug exposure. As shown in Figure 7A, the FI ratios obtained from in vitro flow cytometry data were significantly increased in the cancer cells by the drugs. The increased FI ratio induced by the drug exposure ranged from 1.2 to 6.2 (median: 3.8) in MCF10A and from 10.9 to 29.2 (median: 18.7) in cancer cell lines MCF7 and MDA‐MB‐231. When we designated a cutoff value for the increased FI ratio as 8, cancer cells and noncancer cells were discriminated at the accuracy of 100%. Furthermore, the cutoff value set in vitro was applicable to cancer cells obtained from the xenograft via FNA: the increased FI ratio (median: 23.0; range: 12.6‐82.1) was clearly higher than 8 (Figure 7B), distinguishing between cancer and noncancer cells.

## 4 DISCUSSION

The 5‐ALA–induced PpIX‐fluorescence measurement could become a promising strategy for identification of cancer in clinical practice. However, this method has not been established for breast cancer, and is only available for the brain and urinary bladder,^12^, ^19^, ^20^ with oral^12^ and transurethral^31^, ^32^ administration of 5‐ALA, respectively. Unlike the hollow organ neoplasms, it is next to impossible to topically apply 5‐ALA in breast cancer, and preliminary clinical studies did not necessarily show consistent results by systemic administration of 5‐ALA per os.^13^, ^21^ This is presumably due to intracellular metabolism via its influx/efflux^28^, ^29^, ^30^ and unknown drug delivery efficiency. The cancer microenvironment, including the relatively lower pH and hypoxic conditions of malignant tumors^33^ may also influence 5‐ALA delivery and intracellular PpIX kinetics. In practice, 5‐ALA–induced PpIX‐fluorescence was reportedly suppressed during hypoxic or acidic conditions.^34^, ^35^, ^36^, ^37^ Immature development of tumor vasculatures expressing high multidrug resistance genes^38^ may also influence the efficiency of 5‐ALA delivery to cancer cells. Autofluorescence of cells/tissues may be another potential factor influencing PpIX‐fluorescence.^25^, ^39^, ^40^

The present study revealed breast cancer cell lines MCF7 and MDA‐MB‐231 directly exposed to 5‐ALA exhibit significantly higher PpIX‐FI compared to the noncancer cell line MCF10A. Direct exposure of cells to 5‐ALA ex vivo may be advantageous over oral, systemic, or in situ administration for the following reasons. First, the present method can evaluate differences in PpIX‐FI by comparison of the cells before and after or with and without drug exposure, whereas in situ drug administration does not permit such evaluations. Second, the cancer microenvironment (eg, low pH, hypoxia, and insufficient drug delivery due to the vascular immaturity of the cancer),^41^ would be minimized in ex vivo cells or specimens. Finally, the influence of autofluorescence may be negated in the post ex vivo observation of PpIX‐FI compared with the fluorescence measurements in situ, especially by the subtraction of intracellular FI before 5‐ALA from that after 5‐ALA. Recently, similar experimental approaches were applied to a clinical trial, that is, directly exposing 5‐ALA to urinary cytology. The results showed that direct 5‐ALA exposure is efficient in discriminating between cancer and noncancer cells.^40^, ^42^

According to previous reports, ABCG2 plays an important role in the accumulation of 5‐ALA‐PpIX in cancer cells.^28^, ^29^, ^30^ In the present study, we have clearly demonstrated that combined exposure to 5‐ALA and Ko143 significantly augmented overall PpIX‐FI and reduced cell‐to‐cell variability in breast cancer cell lines. Because of the relatively higher expression of the drug efflux transporter ABCG2 in cancer cells than in noncancer cells, Ko143 was found to alleviate cell‐to‐cell variability in PpIX‐FI. In practice, 5‐ALA combined with ABCG2 inhibitor treatment reduces cell‐to‐cell variability in PpIX‐fluorescence and enhances PpIX accumulation.^42^ In contrast, combined administration of 5‐ALA and Ko143 barely altered the PpIX‐FI in the noncancer cells MCF10A expressing low levels of the ABCG2 transporter. Of note, the ABCG2 inhibitor Ko143, which is currently not approved for clinical administration, would be available only to ex vivo laboratory settings. Thus, comparing PpIX‐FI, with and without combined exposure to 5‐ALA and Ko143, may be applicable to ex vivo discrimination of cancer cells. This ABCG2 inhibitor has recently been used in experimental photodynamic diagnosis and therapy.^31^, ^43^

We measured PpIX‐FI by two modalities, that is, confocal fluorescent microscopy and flow cytometry. The results obtained were somewhat different from those obtained from confocal measurements for PpIX‐FI; the PpIX‐FI in flow cytometry was significantly increased in cancer as well as noncancer cells by 5‐ALA. We assume the different results obtained from these two methods can be explained by the differences in the cells measured. Considering that flow cytometry analyzes overall cells in re‐suspension with no information of morphology and viability of individual cells, this sophisticated modality may have measured the FI not only in viable cells but also in dying or dead cells, the latter of which should express high FI. On the other hand, confocal fluorescence measurements are conducted under direct observations of cell shape; therefore, under confirmation of the cells being viable. On fluorescent measurements, we should consider cell thickness, which may influence the PpIX‐FI; the thicker the cell, the higher the FI. According to our preliminary measurement of cell thickness, cancer cells were thicker than noncancer cells (24 ± 2 μm for MCF7, 33 ± 3 μm for MDA‐MB‐231, and 14 ± 2 μm for MCF10A, n = 6 for each). Considering the large confocal aperture, that is, low confocality of our fluorescence measurement system, the utmost threefold higher FI in cancer cells than in noncancer cells could be explained by cell thickness; however, a much higher FI (>10 fold: see Figures 1 and 2) after 5‐ALA administration in cancer cells would not be explained by the thickness.

The present study also assessed the applicability of PpIX‐FI to cancer delineation using a mouse xenograft of MDA‐MB‐231 by setting a cutoff value designed to mimic FNA in breast tissue. We confirmed that the EGFP‐expressing cancer cells MDA‐MB‐231 showed PpIX‐fluorescence after exposure to 5‐ALA. In contrast, PpIX‐fluorescence was absent in non‐EGFP‐expressing cells obtained from the cancer xenograft, as evidenced by the flow cytometric analysis. In this series of experiments, we only used MDA‐MB‐231 because we found that no adequate size of xenograft tumor was obtained for MCF7 due to the high estrogen dependence for tumor growth.^44^ Since the primary goal of this study was to establish a basic protocol for fluorescence discrimination of breast cancer cells ex vivo by direct exposure to 5‐ALA, we decided not to pursue further examination on the MCF7 xenograft.

There are a few limitations in this study. Firstly, we compared only two breast cancer cell lines with one noncancer cell line. Although these cell lines may represent common types of human breast cancer cells,^45^ the applicability of this method to human breast cancer cells ex vivo remains unknown. It is also uncertain whether or not the ABCG2 inhibitor is invariably essential for PpIX‐based discrimination of human breast cancer cells. Secondly, there is no consideration of the heterogeneity of cancer cells per se, which may also influence the discriminability of cancer cells based on their efflux/influx systems. Finally, prolonged exposure to 5‐ALA may be cytotoxic at concentrations relevant to our experimental conditions, for example, incubation time and dosage,^46^ despite the absence of discernible evidence of cell damage in the present investigation. Despite these limitations, our present study indicates that combined exposure to 5‐ALA and Ko143 may be useful for discrimination of breast cancer cell lines.

Collectively, the present study provides a promising strategy for the discrimination of cancer vs the conventional oral administration of 5‐ALA. Applicability of this strategy to human cancer cells obtained from FNA is an objective of future research. In conclusion, direct exposure to 5‐ALA and the ABCG2 inhibitor Ko143 may be the first step toward realizing the utility of fluorescence‐based breast cancer detection by FNA.

## CONFLICT OF INTEREST

Tetsuro Takamatsu received research funding from USHIO Inc. The remaining authors declare no conflict of interest for this article.
