# Supplementary figures and images for: Fluorescence‐based discrimination of breast cancer cells by direct exposure to 5‐aminolevulinic acid
Source: Cancer Med. 2019 Aug 5;8(12):5524–33. doi: 10.1002/cam4.2466 (PMC6746108; doi:10.1002/cam4.2466)

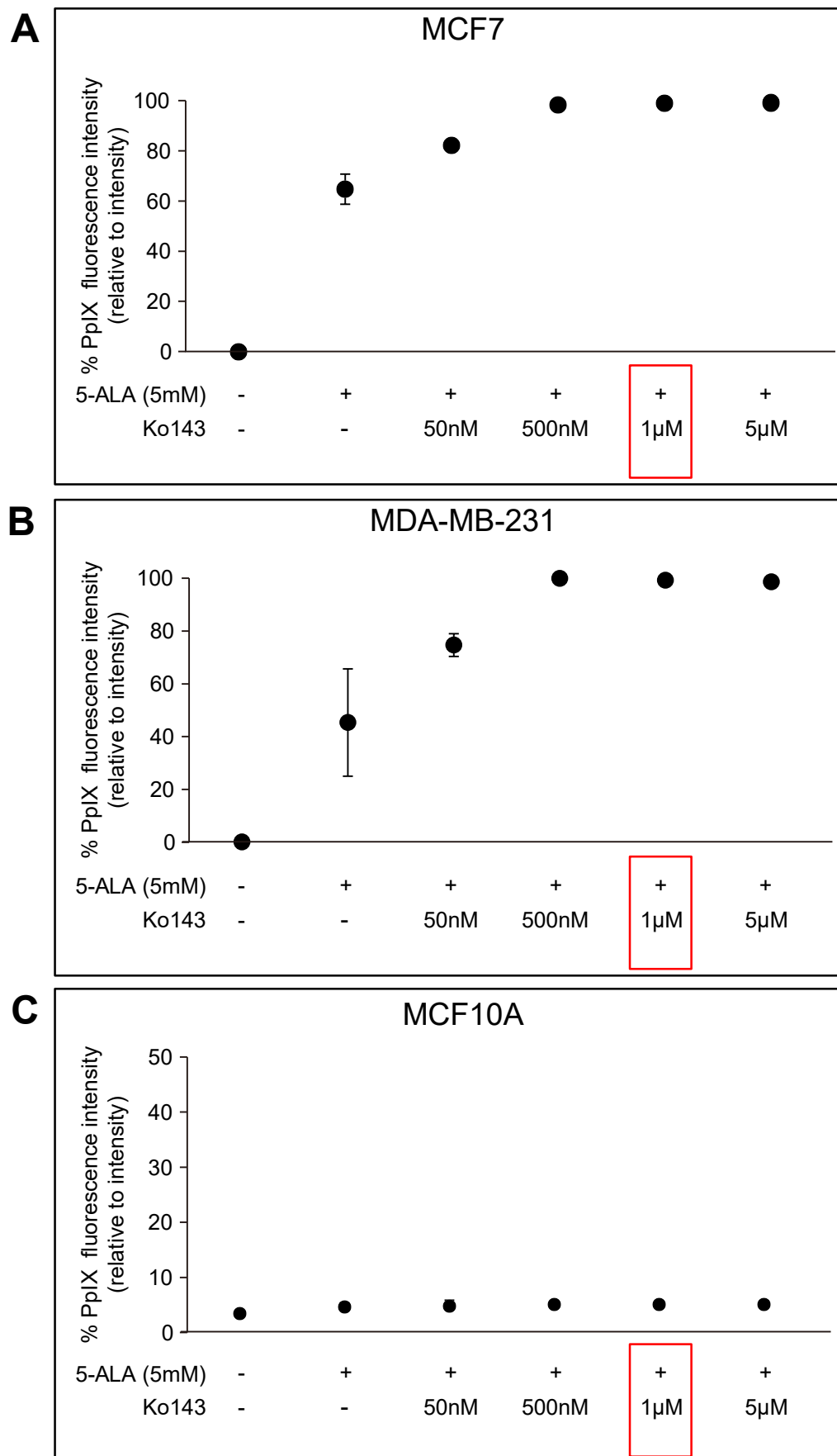

Fig.S1

Supplement: Supplementary file 1 [file CAM4-8-5524-s001.pdf]

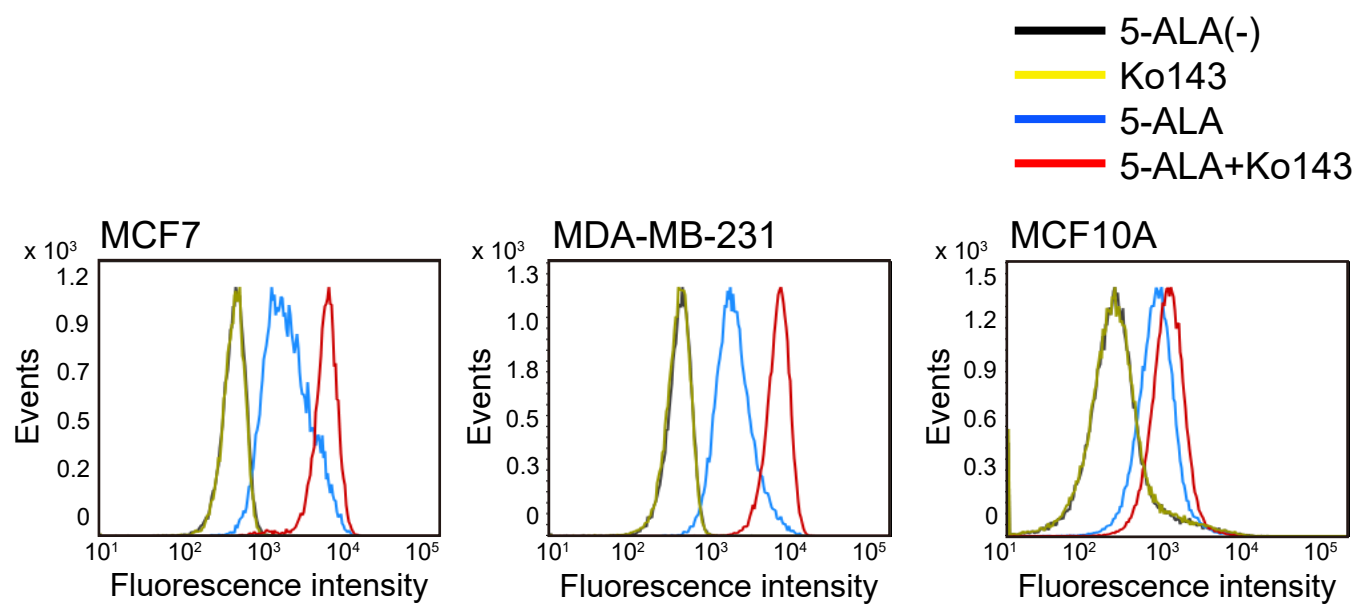

**Fig. S2**

Supplement: Supplementary file 2 [file CAM4-8-5524-s002.pdf]
